# Supplementary figures and images for: Tomato Spotted Wilt Virus Benefits Its Thrips Vector by Modulating Metabolic and Plant Defense Pathways in Tomato
Source: Front Plant Sci. 2020 Dec 18;11:575564. doi: 10.3389/fpls.2020.575564 (PMC7793759; doi:10.3389/fpls.2020.575564)

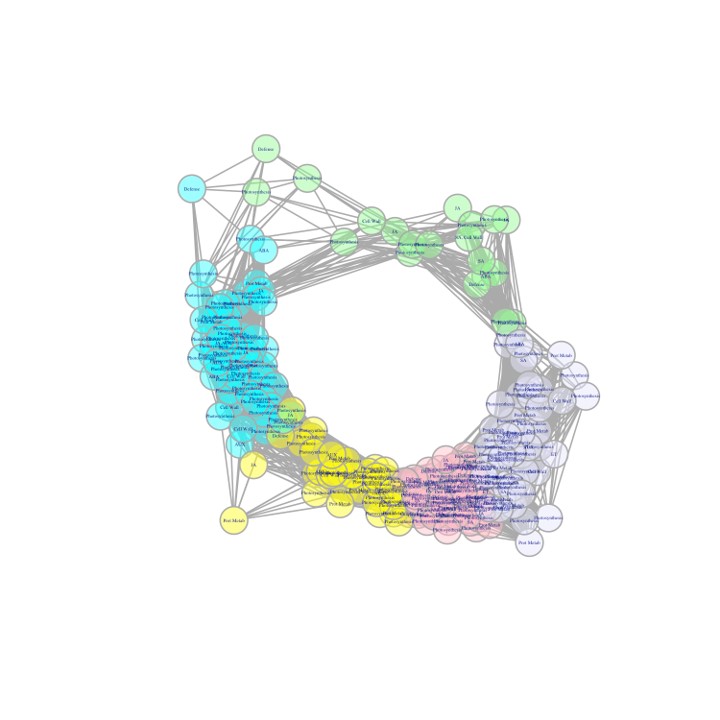

Supplement: Supplementary Figure 1 — Weighted gene co-expression network (WGCNA) plotted using the ‘layout_with_fr’ layout in igraph. Each node represents a probe set id (sequence) from the gene chip. The five clusters of nodes following leading eigen community detection are illustrated by the different colored nodes. The membership of nodes in each community is provided in Supplementary Table 10. [file Image_1.JPEG]
